# Laparoscopic partial splenectomy for traumatic splenic rupture patients is safe and feasible

**DOI:** 10.3389/fsurg.2025.1704119

**Published:** 2025-11-21

**Authors:** Jingna Xu, Weiguang Zhou, Qiang He, Liang Tao

**Affiliations:** 1 Department of Anesthesiology, Haining City Central Hospital, Haining, China; 2 Department of General Surgery, Haining City Central Hospital, Haining, China

**Keywords:** laparoscopic partial splenectomy, splenic rupture, surgery, treatment, safe

## Abstract

**Background:**

Traumatic splenic rupture is a life-threatening acute abdominal condition. While traditional total splenectomy effectively controls bleeding, it results in the loss of splenic function, increasing the risk of infectious complications and long-term health issues. Laparoscopic partial splenectomy, as a minimally invasive spleen-preserving approach, offers a new treatment option for such patients. However, its technical complexity limits its clinical adoption, requiring further experience accumulation and technical optimization.

**Methods:**

This study retrospectively analyzed the clinical data of 13 patients with traumatic splenic rupture treated between 2018 and 2021 to evaluate the safety, efficacy, and applicability of laparoscopic partial splenectomy. All patients were diagnosed via contrast-enhanced abdominal CT imaging. During surgery, partial splenectomy was performed using splenic artery occlusion combined with anatomical dissection. Perioperative data and postoperative changes in blood parameters were recorded.

**Results:**

The median operative time was 120 min, with a median intraoperative blood loss of 1,500 mL. The residual spleen in all 13 patients maintained good blood supply. Blood tests showed a gradual resolution of postoperative inflammatory responses, with platelet levels returning to normal. Imaging follow-up confirmed improved blood supply to the residual spleen. The median postoperative hospital stay was 14 days, and all patients survived and preserved splenic tissue. One patient developed a minor splenic infarction that resolved, and no patient experienced re-bleeding, overwhelming infection, or other major complications.

**Conclusions:**

Laparoscopic partial splenectomy demonstrates favorable safety and efficacy in patients with traumatic splenic rupture. By optimizing intraoperative vascular control and careful management of the resection site, this procedure not only preserves partial splenic function but also reduces postoperative complications. It provides a reliable minimally invasive surgical option for the treatment of traumatic splenic rupture.

## 1 Introduction

As a vital immune organ, the spleen plays a critical role in regulating blood circulation, immune responses, and hematopoietic cell metabolism (1, 2). However, due to its unique anatomical location and fragile tissue texture, the spleen is prone to injury in blunt or penetrating abdominal trauma, resulting in a high incidence of splenic rupture (3–5). This condition is a potentially life-threatening acute abdomen that demands significant attention due to its propensity to cause hemorrhagic shock and secondary intra-abdominal infections.

Traditionally, the management of splenic rupture has predominantly involved open total splenectomy, which effectively controls bleeding but significantly increases the risk of postoperative infectious complications, hematological abnormalities, and long-term health issues due to the loss of splenic function (6, 7). Consequently, balancing hemostasis while preserving splenic function has emerged as a critical focus in the clinical management of traumatic splenic rupture. With the rapid development of minimally invasive surgical techniques, laparoscopic surgery has gradually been applied to splenic surgeries owing to its advantages of reduced trauma, faster recovery, and fewer complications (8, 9). Specifically, laparoscopic partial splenectomy provides a novel treatment option that balances spleen function preservation with damage control for patients with splenic trauma (10, 11). However, due to the spleen's complex vascular anatomy, the variable morphology of splenic ruptures, and the limited surgical workspace, laparoscopic partial splenectomy is technically challenging and demands a higher level of anatomical understanding and surgical skill (12). Additionally, selecting the appropriate surgical timing based on individual patient conditions, optimizing intraoperative techniques, and minimizing postoperative complications remain pressing issues.

Currently, further studies and case series on laparoscopic partial splenectomy in trauma can facilitate broader adoption of this technique (11, 13, 14). This study retrospectively reviewed and analyzed the clinical data of 13 patients with traumatic splenic rupture who underwent laparoscopic partial splenectomy at our institution. By summarizing key surgical techniques and related clinical experiences, this study aims to assess the safety, efficacy, and applicability of laparoscopic partial splenectomy, providing scientific evidence for the standardized application and promotion of this technique. Furthermore, this study aims to contribute practical data to the existing evidence on spleen-preserving trauma management.

## 2 Materials and methods

### 2.1 Patients

Between January 1, 2018 and December 12, 2021, our hospital performed 107 surgeries for traumatic splenic rupture, including 42 laparoscopic total splenectomies, 40 total splenectomies, 13 laparoscopic partial splenectomies, 9 splenic artery embolizations, and 2 partial splenectomies. In this study, we retrospectively collected clinical data of 13 patients with traumatic splenic rupture who underwent laparoscopic partial splenectomy in our hospital to evaluate the safety and feasibility of this procedure. The severity of splenic injury was classified according to the American Association for the Surgery of Trauma (AAST) spleen injury scale. 13 patients met the inclusion criteria for laparoscopic partial splenectomy (LPS). Inclusion criteria were: (1) hemodynamic stability after initial resuscitation; (2) localized or polar splenic injury (AAST grade I–III) confirmed by contrast-enhanced CT; (3) absence of other life-threatening injuries requiring immediate laparotomy; and (4) informed consent obtained from the patient or family. Exclusion criteria included: (1) hemodynamic instability or failure to respond to resuscitation; (2) extensive splenic rupture (AAST grade IV–V); and (3) severe concomitant abdominal or systemic injuries. Total splenectomy was performed in patients with extensive hilar or multifocal lacerations (AAST grade IV–V), uncontrollable bleeding, or hemodynamic instability after initial resuscitation. The cohort included 11 males and 2 females, aged 16–72 years. The causes of injury included falls (including high-level falls), motor vehicle accidents, and crush injuries, all presenting with varying degrees of thoracoabdominal pain. Two patients had a history of diabetes and lumbar spine surgery, while the others had no significant medical or surgical history. None of the patients had a history of upper abdominal surgery or cardiovascular disease. The diagnosis, injury location, and extent of splenic rupture were confirmed by contrast-enhanced abdominal CT combined with diagnostic peritoneal puncture. We retrospectively analyzed perioperative data. Splenic preservation and potential regeneration were assessed by comparing laboratory results from postoperative day 1 to those at discharge, and by evaluating follow-up CT scans of the residual spleen.

### 2.2 Surgical procedure

**Figure 1:**
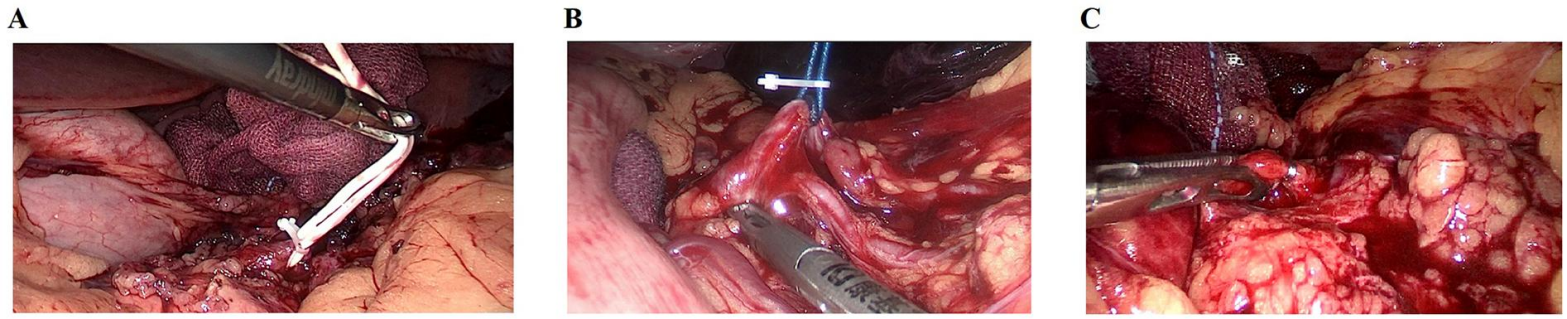
Splenic artery blockade approach. **(A)** Rubber glove ring; **(B)** vascular ribbon; **(C)** metal titanium clips.

A urinary catheter and nasogastric tube were placed preoperatively, and all patients underwent general anesthesia with endotracheal intubation. The right lateral decubitus position was adopted. A laparoscopic port was placed 2–4 cm above and to the left of the umbilicus, with additional ports positioned under the xiphoid, along the right midclavicular line, and at the right anterior axillary line at the umbilical level (or higher in obese patients). The procedure began at the inferior pole of the spleen, where the splenorenal ligament was incised using an ultrasonic scalpel, while carefully avoiding injury to the gastroepiploic vascular arc. The pancreatic tail was exposed, and the main splenic artery was isolated above the pancreatic tail. Temporary occlusion of the main splenic artery was performed using a vascular clamp to reduce splenic perfusion and identify the ischemic demarcation line, followed by selective dissection of segmental vessels supplying the injured pole. Splenic artery occlusion was achieved using a blocking band or metallic titanium clips (Figure 1).

After aspirating perisplenic stagnant blood and thoroughly evaluating the extent of splenic injury, patients with isolated upper or lower pole injuries underwent further dissection of splenic arterial branches. Only patients with localized (AAST grade II–III) upper or lower pole injuries and hemodynamic stability proceeded to partial splenectomy. The injured arterial and venous branches were clipped, and the main splenic artery occlusion was released. Partial splenectomy was performed along the ischemic demarcation line. Hemostasis of the residual spleen surface was achieved with bipolar coagulation and prolene sutures. The anatomical structure of the splenic vessels was meticulously examined to ensure complete ligation of the branches on the resected side. A thorough evaluation of other abdominal organs confirmed no additional injuries. The abdominal cavity was irrigated extensively, and autologous blood was reinfused. The spleen specimen was placed in a retrieval bag and removed. A drainage tube was placed before concluding the surgery. Postoperative outcomes were monitored for complications such as bleeding, infection, thrombosis, and splenic infarction.

## 3 Results

### 3.1 Demographics and preoperative data

Between January 1, 2018 and December 12, 2021, 13 patients with traumatic splenic rupture underwent laparoscopic partial splenectomy. Causes of injury included falls, motor vehicle accidents, crush injuries, and high-altitude falls, with all patients presenting varying degrees of thoracoabdominal pain. Detailed demographic and clinical characteristics are summarized in Table 1. Initial abdominal CT scans identified splenic rupture or contusion, with the primary cause being motor vehicle accidents, followed by crush and high-altitude injuries. The majority of patients were male, with a median age of 43 years (range: 16–72 years). Upper pole splenic ruptures occurred in 6 of 13 cases (46%), while lower pole ruptures occurred in 7 of 13 cases (53%). According to the AAST spleen injury scale, 5 patients (38%) had grade II injuries and 8 patients (62%) had grade III injuries. All injuries were localized to one pole of the spleen and were hemodynamically stable, meeting the indications for partial splenectomy. All 13 laparoscopic partial splenectomies were successfully completed without conversion to open surgery or procedure abortion.

**Table 1:** Demographic and preoperative data.

Case	Sex	Age (years)	Injury causes	Past medical history	Diagnosis	Extent of resection
Case 1	M	41	Fall injury	None	Splenic rupture	Upper pole
Case 2	M	31	Fall injury	None	Splenic contusion	Lower pole
Case 3	M	52	High falling	None	Splenic rupture	Upper pole
Case 4	M	40	High falling	None	Splenic rupture	Upper pole
Case 5	M	59	Traffic accident	Diabetes	Splenic rupture	Lower pole
Case 6	M	59	Traffic accident	None	Splenic contusion	Lower pole
Case 7	M	26	Traffic accident	None	Splenic rupture	Lower pole
Case 8	M	38	Crushing injury	None	Splenic contusion	Upper pole
Case 9	M	43	Crushing injury	None	Splenic rupture	Lower pole
Case 10	F	72	Fall injury	None	Splenic rupture	Upper pole
Case 11	M	16	Traffic accident	None	Splenic rupture	Lower pole
Case 12	F	67	Traffic accident	Lumbar surgery	Splenic contusion	Upper pole
Case 13	M	71	Fall injury	None	Splenic rupture	Lower pole

### 3.2 Perioperative analysis

As shown in Table 2, the median operative time was 120 min (range: 90–180 min), with an estimated median blood loss of 1,500 mL (range: 200–3,000 mL). 8 patients required intraoperative blood transfusions (median 1,000 mL), and no patient required postoperative transfusion. No transfusion-related complications occurred during hospitalization. Two patients (Cases 1 and 2) were transferred to other hospitals 3 and 9 days postoperatively for specialized management of associated injuries. Intraoperatively, three patients experienced active bleeding from the residual splenic surface despite bipolar coagulation. Intraoperative bleeding occurred in 3 patients due to small venous branches near the splenic hilum, all of which were effectively controlled by laparoscopic suturing. The mean drainage volume on postoperative day 1 was 150 mL. No postoperative bleeding or pancreatic fistula was observed. One case of localized splenic infarction was noted, with improved splenic perfusion confirmed by contrast-enhanced CT 30 days postoperatively. No transfusion-related complications occurred during hospitalization. The median postoperative hospital stay was 14 days (range: 10–27 days). And the postoperative residual splenic volume of 13 patients ranged from 19.6 to 238.6 cm^3^, with a median volume of 74.93 cm^3^. These data demonstrate that a substantial portion of splenic tissue was preserved after partial splenectomy, supporting the maintenance of splenic function following surgery. In addition, the relatively wide range of residual volume reflects individual variability in the extent of resection depending on the injury site and intraoperative bleeding control. In short, all patients survived and preserved splenic tissue. One patient developed a minor splenic infarction that resolved, and no patient experienced re-bleeding, overwhelming infection, or other major complications.

**Table 2:** Perioperative data.

Case	Operative time (min)	Blood loss (mL)	Conversion	Transfusion (mL)	Postoperative stay (days)	Postoperative spleen volume (cm^3^)
Case 1	110	2,000	None	1,220	3	238.56
Case 2	160	1,500	None	2,170	9	180.00
Case 3	100	1,800	None	0	13	46.38
Case 4	90	500	None	610	27	74.93
Case 5	120	1,000	None	1,370	20	24.47
Case 6	180	2,000	None	0	23	187.20
Case 7	150	3,000	None	560	10	120.00
Case 8	180	2,000	None	380	11	119.14
Case 9	160	1,000	None	0	15	51.84
Case 10	120	300	None	0	13	79.20
Case 11	120	1,500	None	1,210	14	38.85
Case 12	150	1,000	None	790	14	19.57
Case 13	90	200	None	0	16	43.76

### 3.3 Analysis of hematological parameters

Specific cases were analyzed involving routine blood data as shown in Table 3. Postoperative blood test analysis revealed a significant increase in white blood cell (WBC) counts, particularly on postoperative day 1. For instance, Case 1's WBC count increased from 12.5 × 10⁹/L preoperatively to 22.1 × 10⁹/L, while Case 5's WBC count rose from 6.4 × 10⁹/L to 10.9 × 10⁹/L. This reflected a typical postoperative inflammatory response, which gradually subsided prior to discharge, with most patients' WBC counts approaching or falling below preoperative levels (e.g., Case 11: 7.1 × 10⁹/L at discharge, Case 9: 5.0 × 10⁹/L).

**Table 3:** Blood routine data.

Case	Preoperative			1 day after surgery			Before discharge		
	WBC (L^−1^) × 10^9^	Hb (g·L^−1^)	PLT (L^−1^) × 10^9^	WBC (L^−1^) × 10^9^	Hb (g·L^−1^)	PLT (L^−1^) × 10^9^	WBC (L^−1^) × 10^9^	Hb (g·L^−1^)	PLT (L^−1^) × 10^9^
Case 1	12.5	124	153	22.1	119	294	14.2	93	146
Case 2	30.1	145	265	14.1	101	103	14.4	118	318
Case 3	15.5	95	130	12.2	83	119	8.1	91	428
Case 4	31.5	145	149	14.1	111	94	7.9	133	278
Case 5	6.4	114	207	10.9	108	121	7.3	90	407
Case 6	13.7	143	262	14.1	130	137	8.3	119	558
Case 7	18.4	163	176	18.7	140	126	12.7	142	434
Case 8	5.9	133	128	13.1	121	109	6.1	128	247
Case 9	9.9	129	219	8.4	114	199	5.0	108	483
Case 10	21.3	119	197	12.2	93	64	9.5	104	393
Case 11	10.4	153	274	14.7	103	177	7.1	127	1,071
Case 12	22.9	117	161	13.9	111	150	6.3	101	575
Case 13	12.0	126	222	14.1	113	232	6.5	113	305

WBC, White blood cell; Hb, Hemoglobin; PLT, Platelet.

Hemoglobin (Hb) levels showed relatively minor fluctuations, with slight decreases on postoperative day 1, likely due to intraoperative blood loss and postoperative fluid resuscitation. For instance, Case 2's Hb decreased from 145 g/L preoperatively to 101 g/L, and Case 5's Hb decreased from 114 g/L to 108 g/L. However, some cases exhibited more pronounced declines, such as Case 3 (95 g/L preoperatively to 83 g/L postoperatively).

Platelet counts (PLT) initially decreased postoperatively but subsequently rebounded significantly before discharge. For instance, Case 7's PLT decreased from 176 × 10⁹/L preoperatively to 126 × 10⁹/L on postoperative day 1, then increased to 434 × 10⁹/L before discharge. Some cases showed dramatic PLT increases before discharge, such as Case 11 (274 × 10⁹/L preoperatively to 1,071 × 10⁹/L at discharge).

## 4 Discussion

The spleen, as the largest secondary lymphoid organ in the human body, plays a vital role in various physiological processes, which becomes evident from the complications following total splenectomy (15, 16). Among these complications, the incidence of overwhelming infections can reach as high as 44%, with a mortality rate ranging from 50% to 80%. Studies suggest that preserving at least 25% of splenic tissue with adequate perfusion can maintain normal splenic function (17, 18). Consequently, compared to total splenectomy, partial splenectomy offers significant advantages in preserving postoperative immune function, making it an important focus of development in splenic surgery (19–21). With advancements in minimally invasive techniques, laparoscopic surgery has gained increasing application in managing traumatic splenic rupture (22). It not only allows precise assessment of splenic injuries but also facilitates spleen-preserving procedures in hemodynamically stable patients. However, the high cost of equipment required for interventional treatments limits their availability in primary healthcare settings, highlighting the critical role of laparoscopic partial splenectomy under certain circumstances. Among the 107 patients with traumatic splenic injury treated during the study period, 13 (12%) underwent laparoscopic partial splenectomy. 42 (39%) patients underwent total splenectomy due to high-grade (AAST IV–V) or multifocal injuries and unstable vital signs, which remain the main indications for splenectomy in trauma management. The relatively low proportion of LPS reflects both the strict selection criteria and the technical complexity of the procedure (23). In our trauma center, non-operative management or transcatheter arterial embolization (TAE) is preferred for low-grade injuries when bleeding can be controlled and follow-up imaging is feasible. However, in patients with localized parenchymal rupture and active bleeding confined to one pole, LPS provides direct hemostasis and allows splenic preservation while avoiding potential complications of embolization, such as splenic infarction or abscess formation. Therefore, LPS was selected for hemodynamically stable patients with Grade II–III polar injuries who required surgical control of bleeding. In China and internationally, laparoscopic spleen-preserving techniques have been increasingly adopted for selected trauma cases, yet their application remains limited to specialized centers with advanced laparoscopic experience (24, 25). Previous reports have confirmed that LPS can achieve comparable hemostasis to open surgery while preserving immune function when applied appropriately (26). Our findings are consistent with this growing trend, providing additional real-world evidence that LPS is technically feasible and safe in carefully selected trauma patients.

With the establishment of detailed anatomic understanding of splenic segmental structures and avascular planes since the 1960s–1970s, partial splenectomy has become a technically feasible procedure (27, 28). This anatomical feature provides the theoretical basis for partial splenectomy. The primary role of the splenic artery in supplying blood to the spleen underscores its importance in controlling intraoperative bleeding (29, 30). Additionally, the presence of polar vessels supports the perfusion of the remaining spleen following detachment of the splenic hilum. Our case series demonstrates that combining splenic artery occlusion with secondary hilar dissection effectively reduces postoperative complications and enhances surgical precision. Furthermore, anatomical studies of omental vessels provide a solid foundation for preserving omental blood supply during partial splenectomy, which significantly reduces the risk of postoperative complications while maintaining splenic function.

Key surgical steps in laparoscopic partial splenectomy for traumatic splenic rupture include approach selection, vascular management, resection margin determination, and wound surface management (31, 32). Unlike total splenectomy, partial splenectomy requires special attention to preserving the blood supply to the residual spleen to avoid postoperative infarction or congestion (33, 34). During surgery, occluding the main splenic artery facilitates exploration of splenic injuries. Selective ligation of branch vessels based on segmental vascular anatomy helps identify ischemic lines and guides the resection margin. However, dissecting splenic artery branches can be challenging due to blood and oozing from the wound surface. In such cases, gradual division of the splenic hilum along the estimated ischemic line ensures the integrity of vascular branches. Special care should also be taken when handling omental vessels, particularly during lower pole resection, as preserving the short gastric vessels significantly improves the quality of blood supply to the remaining spleen.

**Figure 2:**
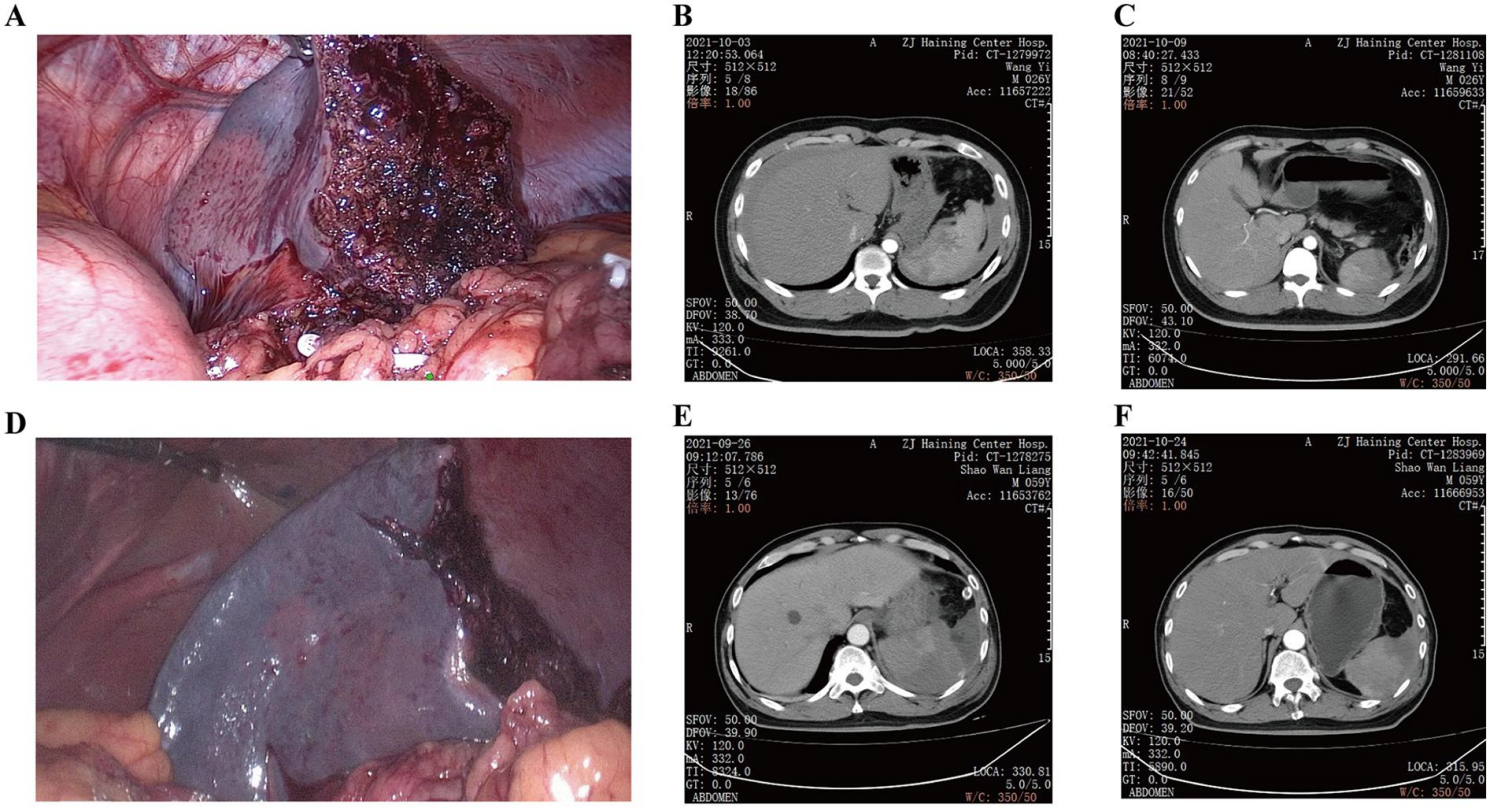
Cases of partial splenectomy with preserved omental blood supply. **(A)** Post-excision trauma with slight ischemic manifestation of the stump; **(B)** pre-operative enhanced CT suggesting injury of the splenic hilum; **(C)** post-operative CT on the 6th day, suggesting a good blood supply of the residual spleen; **(D)** partial resection of the residual spleen with dark blood color and ischemic manifestation; **(E)** re-examination of CT on the 7th day after the operation suggesting a partial ischemic manifestation of the residual spleen; **(F)** re-examination of the CT on the 30^th^ day after the operation. CT, suggesting improvement of ischemia and partial liquefaction of the residual spleen.

In this study, two patients with lower pole injuries extending to the splenic hilum underwent lower pole resection. In one case, persistent bleeding from the wound surface after suturing was controlled by occluding the main splenic artery while preserving short gastric vessel supply, achieving splenic function preservation. On postoperative day six, enhanced CT confirmed good splenic perfusion in one patient (Figures 2A–C). In another case, enhanced CT on postoperative day seven revealed partial ischemia around the wound surface, which improved by day 30, though the patient experienced left upper abdominal pain during hospitalization (Figures 2D–F). It is particularly important to preserve the integrity of the splenic vein trunk during such procedures (35–37). Compared to Warshaw's spleen-preserving technique, preserving venous outflow reduces the risk of wound surface bleeding and prevents long-term complications such as gastric fundus varices (38, 39). By incorporating omental blood supply preservation, this approach achieves both hemostasis and functional preservation of the spleen, as confirmed by postoperative imaging showing satisfactory residual splenic perfusion in all cases.

Key factors in partial splenectomy also include wound surface management and prevention of postoperative complications (11, 40). Bipolar coagulation, figure-8 stitch, and argon beam coagulation are effective techniques for achieving hemostasis and minimizing the risk of bleeding (41, 42). In our series, no patient experienced postoperative bleeding, highlighting the importance of precise surgical techniques and intraoperative management. Additionally, using metal clips for splenic artery occlusion provides excellent hemostatic outcomes while avoiding surgical complications caused by accidental vessel ligation (43). The unique nature of traumatic splenic rupture demands higher surgical precision, especially in emergency settings, requiring efficient team collaboration and robust intraoperative support. Open partial splenectomy remains a viable option in many trauma settings. Compared to traditional open surgery, laparoscopic techniques offer advantages such as enhanced visualization and reduced manipulation of the spleen, enabling successful spleen-preserving procedures in hemodynamically stable patients (11, 24, 44). This study demonstrates the feasibility of laparoscopic partial splenectomy in selected trauma patients. However, as this is a retrospective case series with a small sample size and no control group, selection bias and confounding factors may exist. Future studies with larger, multicenter prospective studies are needed to further confirm the advantages of this technique.

## 5 Conclusion

Laparoscopic partial splenectomy not only demonstrates the minimally invasive advantages in managing traumatic splenic rupture but also effectively avoids severe complications such as thrombocytosis and venous thrombosis by preserving splenic function. Unlike interventional treatments, this technique allows resection of ischemic splenic tissue, preventing postoperative splenic infarction and representing a promising direction in splenic surgery. With continuous improvements in surgical techniques and supportive equipment, laparoscopic partial splenectomy is expected to play an increasingly vital role in the treatment of traumatic splenic rupture, providing better prognoses and improved quality of life for patients.

## Data Availability

The original contributions presented in the study are included in the article/Supplementary Material, further inquiries can be directed to the corresponding author.
